# SPMSQ for risk stratification of older patients in the emergency department: An exploratory prospective cohort study

**DOI:** 10.1007/s00391-019-01626-z

**Published:** 2019-10-16

**Authors:** A. Schönstein, H.-W. Wahl, H. A. Katus, A. Bahrmann

**Affiliations:** 1 Network Aging Research, Heidelberg University, Heidelberg, Germany; 2 Heidelberg University Hospital, Heidelberg, Germany

**Keywords:** Cognition, Geriatrics, Screening, Adverse outcomes, Mortality, Kognition, Geriatrie, Screening, Adverse Outcomes, Mortalität

## Abstract

**Background:**

Risk stratification of older patients in the emergency department (ED) is seen as a promising and efficient solution for handling the increase in demand for geriatric emergency medicine. Previously, the predictive validity of commonly used tools for risk stratification, such as the identification of seniors at risk (ISAR), have found only limited evidence in German geriatric patient samples. Given that the adverse outcomes in question, such as rehospitalization, nursing home admission and mortality, are substantially associated with cognitive impairment, the potential of the short portable mental status questionnaire (SPMSQ) as a tool for risk stratification of older ED patients was investigated.

**Objective:**

To estimate the predictive validity of the SPMSQ for a composite endpoint of adverse events (e.g. rehospitalization, nursing home admission and mortality).

**Method:**

This was a prospective cohort study with 260 patients aged 70 years and above, recruited in a cardiology ED. Patients with a likely life-expectancy below 24 h were excluded. Follow-up examinations were conducted at 1, 3, 6 and 12 month(s) after recruitment.

**Results:**

The SPMSQ was found to be a significant predictor of adverse outcomes not at 1 month (area under the curve, AUC 0.55, 95% confidence interval, CI 0.46–0.63) but at 3 months (AUC 0.61, 95% CI 0.54–0.68), 6 months (AUC 0.63, 95% CI 0.56–0.70) and 12 months (AUC 0.63, 95% CI 0.56–0.70) after initial contact.

**Conclusion:**

For longer periods of observation the SPMSQ can be a predictor of a composite endpoint of adverse outcomes even when controlled for a range of confounders. Its characteristics, specifically the low sensitivity, make it unsuitable as an accurate risk stratification tool on its own.

**Electronic supplementary material:**

The online version of this article (10.1007/s00391-019-01626-z) contains supplementary material, which is available to authorized users.

## Zusammenfassung

**Hintergrund:**

Die Risikostratifizierung von älteren Patienten in der Notaufnahme gilt als vielversprechender und effizienter Lösungsansatz, um die steigende Nachfrage nach geriatrischer Notfallmedizin zu bewältigen. Bisher zeigte sich die prädiktive Validität des am häufigsten eingesetzten Instruments, dem Identification of Seniors at Risk (ISAR), für deutsche Stichproben jedoch als begrenzt. Da die interessierenden Outcomes, wie Rehospitalisierung, Pflegeheimübersiedlung und Mortalität deutlich mit kognitiver Beeinträchtigung zusammenhängen, war es unser Ziel, das Potenzial des Short Portable Mental Status Questionnaire (SPMSQ) als Instrument zur Risikostratifizierung von älteren Notaufnahmepatienten zu überprüfen.

**Fragestellung:**

Schätzung der prädiktiven Validität des SPMSQ für einen kombinierten Endpunkt adverser Outcomes (Rehospitalisierung, Pflegeheimübersiedlung und Mortalität).

**Daten und Methode:**

Es handelte sich um eine prospektive Kohortenstudie mit 260 Patienten im Alter von mindestens 70 Jahren, die in einer kardiologischen Notaufnahme rekrutiert worden waren. Patienten mit einer Lebenserwartung von unter 24 h wurden exkludiert. Follow-ups fanden nach 1, 3, 6, und 12 Monaten statt.

**Ergebnisse:**

SPMSQ war signifikanter Prädiktor für den kombinierten Endpunkt adverser Outcomes zwar nicht für 1 Monat (AUC: 0,55; 95 % KI 0,46–0,63), aber für 3 Monate (AUC: 0,61; 95 % KI 0,54–0,68), 6 Monate (AUC: 0,63; 95 % KI 0,56–0,70) und 12 Monate (AUC: 0,63; 95 % KI 0,56–0,70) nach Erstkontakt.

**Schlussfolgerung:**

Für längere Beobachtungszeiträume scheint der SPMSQ, auch unter Kontrolle potenziell konfundierender Variablen, ein Prädiktor für adverse Outcomes zu sein. Seine Eigenschaften, insbesondere die niedrige Sensitivität, machen ihn jedoch für den Einsatz als alleiniges Screening-Instrument wenig tauglich.

For many older patients the emergency department (ED) is an entry point into the healthcare system. Geriatric emergency medicine is a resource intensive process and with ongoing demographic aging the already high demand is expected to rise even further [1]. At present, special needs of geriatric patients are likely to be overlooked in the ED [26]. To face this challenge and improve pathways towards optimal geriatric healthcare, the geriatric medical concept of the state government of Baden-Württemberg recommends screening older patients for those at high risk for adverse outcomes at the very beginning of the medical treatment, which is often in the ED of acute care hospitals [32].

## Introduction

Fundamentally, risk stratification is intended to be part of a two-step process: first, a screening tool is used for the brief risk stratification of all presenting older patients. Second, those patients that screen positive undergo a multimodal geriatric assessment or some other elaborate diagnostic procedure, which then in consequence enables the clinician to reliably identify the needs of geriatric patients [4, 22]. Risk stratification of older patients in the ED therefore strives to enable the healthcare system to manage its resources as efficiently as possible. Additionally, the goal is to provide the identified high-risk patients with a more thorough diagnostic process than exerted in usual ED care; however, despite a growing body of relevant literature, implementing risk stratification processes targeted at older patients in German EDs seems to fall short [35]. The reasons for this situation include the complex characteristics of the ED setting, ambiguous results about the validity of the potentially useful instruments, as well as the questionable clinical utility.

## Characteristics of the ED setting and risk stratification with identification of seniors at risk (ISAR)

The key to any systematic screening in the ED is feasibility as ED settings provide limited time and room as well as often noisy and busy surroundings. Not only are multimodal geriatric assessments not suited for this environment, some of the screening methods designed specifically for the risk stratification of older adults are likely too long and effortful for efficient use in EDs [14, 36]. In a consensus statement for the identification of geriatric patients in the ED setting in Germany, the German Geriatric Society as well as the German Society of Gerontology and Geriatrics mentioned a number of potential tools for the risk stratification of older adults in the ED setting [6, 16, 21]. Specifically, the use of the identification of seniors at risk screening (ISAR) tool was recommended for settings where no other instruments or geriatric expertise are available, mainly because of the ISAR’s simple administration and its existing extensive body of international literature [35]; however, while positive and negative results on the predictive validity of the ISAR have been reported in the international literature [9, 29], meta-analyses found it to have either insufficient or only modest predictive accuracy [5, 13]. In light of negative results, Hwang and Carpenter argued that while more accurate tools are being developed the ISAR should continue to be used to ensure awareness and understanding of geriatric patients beyond the acute problem [17]. The only study that examined the predictive validity of the ISAR in a German sample of ED patients found it to have “acceptable” predictive validity [30]. There are two major aspects that complicate the integration of the ISAR tool into the clinical routine: first, with the risk of adverse events (e.g. rehospitalization, nursing home admission and mortality) it measures a construct of general risk, which is difficult to grasp and unspecific regarding its medical indications. Second, in the studies conducted using German ED samples, the ISAR classified more than 80% of patients as high-risk patients [30, 37], thus questioning its specificity and ability to strengthen the effective use of resources.

Addressing the previously mentioned concerns and the suggestion made in the literature to explore alternative variables for the risk stratification of older ED patients [5], the objective of this study was to examine the predictive validity of the cognitive screening tool short portable mental status questionnaire (SPMSQ [25]) for adverse events after an ED hospital stay. The SPMSQ is an established short cognitive test that has already found application in the ED setting [28]; it has also been shown to predict adverse events in older patients [21, 31]. Furthermore, cognitive impairment is common but often remains undetected or clinically unused in older ED patients [15]. According to the recommendations of the Society for Academic Emergency Medicine cognitive screening can even be seen as one of the major quality indicators in geriatric emergency medicine [34]. To the best of our knowledge, there is no study that has examined the predictive validity of the SPMSQ for adverse events in a sample of German ED patients across a considerable observational period. Consequently, due to the need for risk stratification and cognitive screening in the ED and the existing strong relationships between cognitive impairment and undesired outcomes, this study examined the suitability of cognitive screening with the SPMSQ as a tool for risk stratification up to a 1-year interval.

## Methods

### Study design and participants

This was a single center, exploratory and prospective cohort study with 260 consecutively recruited ED patients. The data on the predictive validity of the SPMSQ were drawn from the usual care group of an ongoing intervention study. The study was approved by the ethics committee at the medical faculty of Heidelberg University (S-455/2016). Since the study was based on the usual care group of an ongoing intervention study, no specific power calculation was conducted; however, the overall sample size of 260 can be qualified as similar to comparable studies in the existing literature (e.g. [3, 29]).

Recruitment was done by the first author and took place 7 days a week during the day shifts in a cardiological ED (chest pain unit) affiliated with a university hospital, with 12 beds in the ED and a total of 114 beds in the associated cardiology department. The first patient was recruited in July 2017 and the last patient in May 2018. Patients aged 70 years or above were included. Exclusion criteria were missing informed consent or a likely life expectancy of less than 24 h. Due to procedural reasons, patients that had to undergo isolated care were also not included in the study. Patients were asked to participate in the study after the initial medical examination. On agreement, a respective informed consent document to participate in all data waves was signed. Follow-ups were conducted 1, 3, 6 and 12 months after initial contact via telephone interviews. The data were combined with hospital files, online death recording via obituaries and registry office information.

### Measures and outcomes

In addition to several demographic characteristics, the patient’s cognitive performance was assessed by use of the SPMSQ tool, which can be retrieved from the original publication [25] or other available resources [12, 18]. The SPMSQ score is derived from the amount of errors based on a 10-item list by coding errors as “1” and correct answers as “0”. Items include tasks on orientation (“What is the date today?”), memory (“What was your mother’s maiden name?”) and attention (“Subtract 3 from 20 and keep subtracting 3 from each new number, all the way down”). Thus, individual cognitive scores ranged from 0 to 10 errors, with lower values indicating better cognitive performance.

As outcomes unplanned rehospitalizations (ED and general) were recorded as well as nursing home admissions and all-cause mortality. For the primary analysis all outcomes were combined into a binary coded composite adverse outcome variable, meaning at least one of the events had occurred within 1 month after initial contact, if the composite outcome was coded as positive for the first follow-up. This composite outcome was examined for primarily 1 month, but further also for 3, 6 and 12 months after initial contact. For a secondary analysis the all-cause mortality within 1 year after initial contact was also examined.

### Statistical methods

Descriptive statistics of the sample were calculated using means and standard deviations for continuous normally distributed variables, median and interquartile range for continuous/discrete but not normally distributed variables and absolute and relative frequencies for categorical variables. Group differences across these variables were calculated for cognitively impaired and unimpaired patients (SPMSQ error score ≥3 and <3). Given the binary coding of the primary outcome, logistic regression models were used to test for the relationship between the SPMSQ score and the primary outcome. In these analyses, pairwise deletion was used for missing data. Receiver operating characteristic (ROC) curves were used to illustrate the discriminatory performance of the cognitive risk screening. For the secondary outcome, a survival analysis was conducted with Kaplan-Meier estimates. Statistical analyses were performed using SAS Version 9.4 (SAS Institute Inc., Cary, NC, USA).

## Results

Descriptive statistics for the 260 included patients can be found in Table 1. Patients were mostly male 163/260 (63%). The mean age was 79 years (*SD* = 5.97 years), 37/260 (14%) of the patients had no education beyond the basic school level, 156/260 (60%) completed an apprenticeship, 48/260 (19%) finished a university degree and 19/260 (7%) held a PhD. As also displayed in Table 1, using the SPMSQ cut-off of ≥3 errors, patients identified by the SPMSQ as cognitively impaired (60/260 or 23%) were older (*M*_Diff_ = 3.91 years; *t*(91) = 4.44; *p* <0.001) and less educated (*U* = 4264.00; *p* <0.001; *r* = −0.24) than those with a negative SPMSQ result (200/260 or 77%).

**Table 1:** Descriptive statistics of the total sample and group differences between patients classified as normal or impaired by the SPMSQ

Characteristic	Total (*N* = 260)	SPMSQ normal (<3) (*N* = 200)	SPMSQ impaired (≥3) (*N* = 60)	*p*-value
*Age (years)*	79.31 (5.97)	78.40 (5.65)	82.31 (6.07)	**<0.001**
*Sex*				
Male	163 (63%)	130 (65%)	33 (55%)	0.16
Female	97 (37%)	70 (35%)	27 (45%)	
*BMI*	26.84 (4.74)	27.06 (4.49)	26.08 (5.47)	0.21
*CACI*	5 (4–7)	5 (4–7)	6 (5–7.5)	0.12
*Education*				
None	37 (14%)	21 (11%)	16 (27%)	**<0.001**
Apprenticeship	156 (60%)	118 (59%)	38 (63%)	
University degree	48 (19%)	45 (23%)	3 (5%)	
PhD or similar	19 (7%)	16 (8%)	3 (5%)	

Data are number (% of group total), mean (SD), or median (interquartile range)

*p* values for group differences from Welch’s t‑test (age, BMI), Mann-Whitney test (CACI, education) and from χ2-test (sex); significant *p-*values in bold

*BMI* body mass index, *CACI* Charlson age-comorbidity index

Results for logistic regression regarding the composite outcome and related patient attrition are reported in Table 2. The composite endpoint occurred in 64/250 (26%) at 1 month, 117/249 (47%) at 3 months, 145/245 (59%) at 6 months and 165/245 (67%) at 12 months after initial contact. Thus, until 12 months after initial contact 15/260 (6%) patients or indirect follow-ups provided insufficient information on the outcomes for the cases to be included in the analysis. In the univariate logistic regression model SPMSQ was a statistically significant predictor of the composite endpoint at 3 months (odds ratio, OR: 1.34, 95% confidence interval, CI 1.12–1.60), at 6 months (OR: 1.47, 95% CI 1.20–1.80) and at 12 months (OR: 1.54, 95% CI 1.22–1.93) but not at 1 month (OR: 1.13, 95%CI 0.94–1.36) after initial contact. Statistical significance was retained, when controlling for a range of possible confounders (e.g. age, sex, education, body mass index, and comorbidity).

**Table 2:** Univariate and multivariate odds ratios (OR) and 95% confidence intervals (CI) for the composite adverse outcome variable predicted by SPMSQ errors at initial contact

Time after initial contact	*n*	Patients with adverse outcome (*n*, %)	Univariate		Multivariate/adjusted^a^	
			OR	95% CI	OR	95% CI
1 month	250	64 (26%)	1.13	0.94–1.36	1.09	0.90–1.32
3 months	249	117 (47%)	1.34**	1.12–1.60	1.31**	1.09–1.57
6 months	245	145 (59%)	1.47***	1.20–1.80	1.45***	1.18–1.79
12 months	245	165 (67%)	1.54***	1.22–1.93	1.53***	1.20–1.94

^a^This multivariate model was adjusted for patient sex, education and body mass index (BMI) at initial contact. Age and comorbidity at initial contact were also controlled by using the score of the Charlson age-comorbidity index

**p < 0.01, ***p < 0.001

**Fig. 1:**
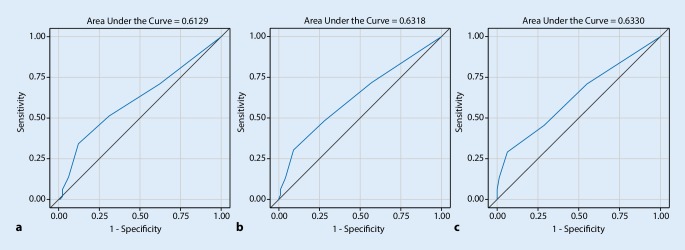
Receiver operating characteristics (ROC) curves for SPMSQ scores as a continuous predictor of the composite outcome at **a** 3 months, **b** 6 months and **c** 12 months after initial contact. Areas under the curve (AUC) were 0.61 (95% CI 0.54–0.68), 0.63 (95% CI 0.56–0.70) and 0.63 (95% CI 0.56–0.70), respectively

Fig. 1 displays the exact discriminatory performance of the SPMSQ score (continuous) for those time points where it was found to be a significant predictor of the composite outcome, hence the 3, 6, and 12-month intervals. Associated areas under the curve (*AUC*) for all time points were 0.55 (95% CI 0.46–0.63) for 1 month, 0.61 (95% CI 0.54–0.68) for 3 months, 0.63 (95% CI 0.56–0.70) for 6 months and 0.63 (95% CI 0.56–0.70) for 12 months after initial contact. Sensitivities and specificities of the SPMSQ for the prediction of the composite outcome across different possible cut-off values can be found in the Supplementary material Table 1. For the time points where SPMSQ was a significant predictor of adverse outcomes, the associated sensitivities and specificities using the ≥3 errors cut-off were as following: 34% sensitivity and 88% specificity (3 months), 30% sensitivity and 91% specificity (6 months) and 29% sensitivity and 94% specificity (12 months).

**Fig. 2:**
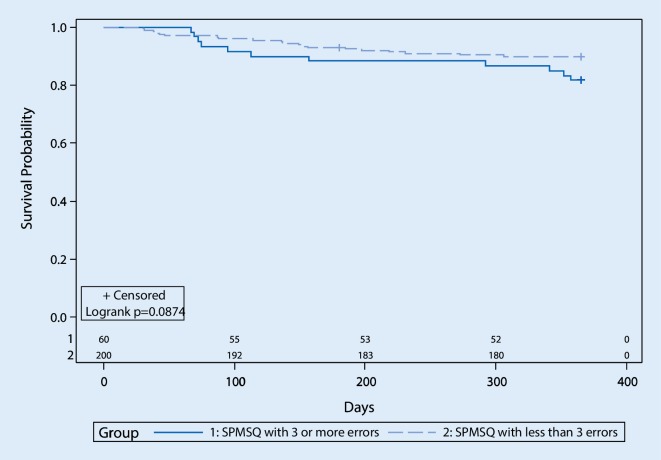
Survival plots for patients classified as cognitively impaired (group 1) or unimpaired (group 2) by the SPMSQ with cut-off ≥3. Numbers above the x-axis indicate the count of patients at risk in the respective groups

Kaplan-Meier curves were used to analyze patient survival probabilities depending on positive or negative SPMSQ results (with cut-off ≥3; see Fig. 2). Of the total sample (*N* = 260) one patient with negative SPMSQ was lost to follow-up at 6 months after initial contact and therefore censored. Overall, the log-rank test showed no statistically significant differences between the resulting two survival curves, although there was a trend that lowered cognitive performance was associated with higher all-cause mortality (χ^2^ (1) = 2.92, *p* = 0.087).

## Discussion

To the best of our knowledge this is the first study that examined the predictive validity of the cognitive screening tool SPMSQ for the risk stratification of older ED patients regarding adverse outcomes (e.g. rehospitalization, nursing home admission, mortality). The key findings can be summarized as following:

- SPMSQ seems to be a useful predictor of adverse outcomes in older German ED patients; however, not for brief (e.g. 1 month) but only for longer observation periods (e.g. 1 year). This relationship remained stable when controlling for a range of confounders.
- The suggested ≥3 errors cut-off appeared to be the most useful when predicting adverse outcomes at different points in time.
- While the specificity is high sensitivity is low. Overall, these characteristics can be regarded as insufficient for use as a screening tool.
- Although a tendency was observed for a decreased 1‑year survival probability of patients with a SPMSQ score of ≥3 errors when compared to those with <3 errors, results were not statistically significant.

Geriatric screening in the ED is of special relevance, because scarcity of resources and increasing demand for geriatric emergency medicine necessitate an empirically tested approach for risk stratification of the patients. Identified high-risk patients can undergo a multimodal assessment and, if suitable profit from specialized interventions or optimized treatment paths for geriatric patients [33]. The best studied instrument and therefore the reference standard for qualifying our results is the ISAR. Singler et al. [30] reported the ISAR to predict adverse outcomes at 28 days and at 6 months after initial contact in a German ED sample. Even though ISAR measures an abstract risk of adverse outcomes and SPMSQ was designed as a cognitive screening tool, cognitive impairment has shown to be substantially related to adverse outcomes, such as rehospitalization, nursing home admittance, and mortality ([11, 21, 31]). For risk stratification purposes, especially the short-term development of patients may be of interest; therefore, the primary analysis used the same composite outcome as the study conducted by Singler et al. [30] and similarly focused on the prediction of adverse events 1 month after initial contact but also for longer observational intervals. For the 1‑month observation period, SPMSQ was not an efficient predictor of adverse outcomes; however, for longer observation periods (3, 6 and 12 months) SPMSQ predicted adverse outcomes even when controlled for a range of confounders, such as patients’ sex, age, comorbidity and body mass index, which may be of interest since the data were collected in a cardiological ED and also education due to potentially protective cognitive reserve [27]. The overall AUC effect size at 6 months was found to be in a comparable magnitude as observed with ISAR. Consequently, the performance of solely going for the SPMSQ seems at first glance to be similar to the ISAR. In addition, the results are in accordance with previously reported findings in the literature that cognitive impairment as measured by the SPMSQ is a predictor of adverse events. This further underlines the usefulness of cognitive measures for risk stratification of older ED patients, which is already considered in existing tools, such as the acutely presenting older patients (APOP) screener [7, 8, 20]; however, limitations of using the SPMSQ as a risk stratification tool in the ED geriatric patient population must be noted as well. Regarding the sensitivity and specificity of the SPMSQ for detecting risk of adverse outcomes, compared to the results reported in the study of Singler et al. [30], the SPMSQ was found to have a higher specificity but a much lower sensitivity than the ISAR for predicting adverse outcomes at 6 months after initial contact. If sensitivity and specificity were weighted equally (e.g. by examining Youden’s *J*), overall diagnostic accuracy of the SPMSQ to predict adverse events would be comparable to that of the ISAR; however, the potential harm from false negatives deserves special consideration. For example, when overlooking a patient with high risk because of a negative SPMSQ categorization and consequently not taking any active measures to prevent the adverse outcome, the consequences would be far more serious than from a false positive. A false positive would only result in extra time spent to conduct a multimodal assessment with a patient that was categorized as a high-risk patient by the ISAR but that is, in reality, at low risk of adverse outcomes. Thus, the SPMSQ appears to be inferior in terms of use as a screening instrument when compared to the results of the ISAR as reported by Singler et al. [30]. Finally, the association of cognitive impairment as categorized by the SPMSQ and the all-cause mortality of the sample of older cardiology ED patients was examined. The results of current research point to cognitive impairment being a clear predictor of mortality [2, 19, 24]. Furthermore, this may be of special relevance in cardiology patients, since cognitive impairment was found to not only be associated with detrimental cardiological events [23], but also other predictors of mortality in cardiological patients, such as malnutrition [10]. Surprisingly, however, no robust relationship between the SPMSQ cut-off and survival was found. This is seen as an important research question for higher powered studies in the future, with possibly longer observation periods.

### Limitations

Several limitations must be considered when interpreting the results. Interviewer bias may be possible because data collection and follow-up were conducted entirely by the first author of this study, who was not blinded regarding the study goal; however, fully standardized measures and objective outcomes were used that are not open to interpretation. Even though this was an exploratory study, multiplicity should also be addressed. The results remained significant when multiplicity was adjusted for by using the established Bonferroni-Holm correction of the alpha significance level. Multiplicity is therefore seen as a relatively minor problem in this analysis. Another possible source of bias is that screening was only possible with patients where an informed consent procedure was feasible. Patients with very severe medical problems were consequently excluded. Since these patients are obviously high-risk patients, they cannot be regarded as the target group for geriatric screening; however, the fact that patients undergoing isolated care could not be included poses a risk to the external validity of the results presented in this article. Additionally, due to mostly conducting follow-ups via phone calls, it was not possible to provide reliable incidences of the outcomes that were combined into the composite outcome separately. For example, the outcome mortality may have masked a previous rehospitalization because it was difficult to retrieve this information. Finally, it must be emphasized that patients were recruited from a university affiliated cardiology ED that may not be representative of the general ED population due to different morbidities and due to its popularity with private patients from different locations. This also can be seen as a risk to the external validity.

### Conclusion

In longer observation periods the cognitive screening tool SPMSQ can be a predictor of adverse outcomes, even when controlled for a range of relevant confounders. Its characteristics, however, specifically the low sensitivity, make it unsuitable as an accurate risk stratification tool alone. Combinations with other risk screening procedures may however be promising.

## Practical conclusion

- The SPMSQ proved to be feasible for use in the ED setting in this sample and was a predictor of adverse outcomes.
- The SPMSQ does not have the capacity to replace risk stratification with common geriatric screening tools like the ISAR.
- Further research into risk stratification with different cognitive screening tools and combinations with other risk stratification devices may produce results with higher sensitivity.

## Caption Electronic Supplementary Material

Supplementary Table 1
