# Supplementary material for: SPMSQ for risk stratification of older patients in the emergency department: An exploratory prospective cohort study
Source: Z Gerontol Geriatr. 2019 Oct 16;52(Suppl 4):222–8. doi: 10.1007/s00391-019-01626-z (PMC6821671; doi:10.1007/s00391-019-01626-z)
Supplement: Supplementary file 1 — Supplementary Table 1 [file 391_2019_1626_MOESM1_ESM.docx]

Supplementary material

Supplementary Table 1

Sensitivity, specificity, and associated Youden’s *J* of SPMSQ for predicting a combined endpoint of adverse outcomes at 3, 6 and 12 months for different cut-offs.

| SPMSQ Cut-Off | Sensitivity | Specificity | Youden’s *J* |
| --- | --- | --- | --- |
| *Time: 3 months after initial contact.* | | | |
| ≥0 | 1 | 0 | 0 |
| ≥1 | 0.71 | 0.38 | 0.09 |
| ≥2 | 0.51 | 0.69 | 0.20 |
| ≥3 | 0.34 | 0.88 | 0.22 |
| ≥4 | 0.14 | 0.94 | 0.08 |
| ≥5 | 0.06 | 0.98 | 0.04 |
| ≥6 | 0.02 | 0.98 | 0 |
| ≥7 | 0 | 0.99 | 0 |
| ≥8 | 0 | 1 | 0 |
| *Time: 6 months after initial contact.* | | | |
| ≥0 | 1 | 0 | 0 |
| ≥1 | 0.72 | 0.43 | 0.15 |
| ≥2 | 0.48 | 0.72 | 0.20 |
| ≥3 | 0.30 | 0.91 | 0.21 |
| ≥4 | 0.13 | 0.96 | 0.09 |
| ≥5 | 0.06 | 0.99 | 0.05 |
| ≥6 | 0.03 | 0.99 | 0.02 |
| ≥7 | 0.01 | 1 | 0.01 |
| ≥8 | 0 | 1 | 0 |
| *Time: 12 months after initial contact.* | | | |
| ≥0 | 1 | 0 | 0 |
| ≥1 | 0.71 | 0.45 | 0.16 |
| ≥2 | 0.46 | 0.71 | 0.17 |
| ≥3 | 0.29 | 0.94 | 0.23 |
| ≥4 | 0.13 | 0.87 | 0.00 |
| ≥5 | 0.06 | 1 | 0.06 |
| ≥6 | 0.03 | 1 | 0.03 |
| ≥7 | 0.01 | 1 | 0.01 |
| ≥8 | 0 | 1 | 0 |

*Note*: Possible range of values for the SPMSQ score is 0 – 10 errors. Sensitivity and Specificity were rounded to two decimals.
